# RACK1 Is a Ribosome Scaffold Protein for β-actin mRNA/ZBP1 Complex

**DOI:** 10.1371/journal.pone.0035034

**Published:** 2012-04-16

**Authors:** Marcello Ceci, Kristy Welshhans, Maria Teresa Ciotti, Rossella Brandi, Chiara Parisi, Francesca Paoletti, Luana Pistillo, Gary J. Bassell, Antonino Cattaneo

**Affiliations:** 1 European Brain Research Institute (EBRI), Rome, Italy; 2 Department of Biological Sciences, Kent State University, Kent, Ohio, United States of America; 3 Institute of Neurobiology and Molecular Medicine-CNR, Rome, Italy; 4 Departments of Cell Biology, Neurology, Emory University School of Medicine, Atlanta, Georgia, United States of America; 5 Scuola Normale Superiore di Pisa, Pisa, Italy; Virginia Commonwealth University, United States of America

## Abstract

In neurons, specific mRNAs are transported in a translationally repressed manner along dendrites or axons by transport ribonucleic-protein complexes called RNA granules. ZBP1 is one RNA binding protein present in transport RNPs, where it transports and represses the translation of cotransported mRNAs, including β-actin mRNA. The release of β-actin mRNA from ZBP1 and its subsequent translation depends on the phosphorylation of ZBP1 by Src kinase, but little is known about how this process is regulated. Here we demonstrate that the ribosomal-associated protein RACK1, another substrate of Src, binds the β-actin mRNA/ZBP1 complex on ribosomes and contributes to the release of β-actin mRNA from ZBP1 and to its translation. We identify the Src binding and phosphorylation site Y246 on RACK1 as the critical site for the binding to the β-actin mRNA/ZBP1 complex. Based on these results we propose RACK1 as a ribosomal scaffold protein for specific mRNA-RBP complexes to tightly regulate the translation of specific mRNAs.

## Introduction

The localization and translation of mRNAs in specific regions of the cell is an evolutionarily conserved mechanism to regulate the quantity of proteins within specific cellular compartments [1]. In neurons, the dendritic localization of mRNAs and subsequent translation at stimulated synapses is believed to be responsible for long term synaptic plasticity [2], [3]. The mRNAs are transported to distal dendrites in a translational silent manner by binding to RNA-binding proteins (RBPs) within specific ribonucleic particles, called transport RNPs. In these particles, the RBPs show a dual function: they act both as mRNA transport factors and as translation repressors. At their destinations, neuronal activity stimulates post-translation modifications of RBPs, which promote the release and translation of associated mRNAs.

The Zipcode binding protein 1 (ZBP1) is one of several RBPs found in RNPs whose translational regulation has been extensively studied. ZBP1 binds a wide variety of mRNAs, but only its binding to 3′UTR of β-actin mRNA has been characterized. During growth cone turning, ZBP1 binds the β-actin mRNA, represses its translation and transports it to growth cones [4]. The phosphorylation of ZBP1 by Src, stimulated by Brain-Derived Neutrophic Factor (BDNF), determines the release and the local translation of β-actin mRNA favoring the growth cone [5], [6].

The local translation of mRNAs at final destinations is fast and tightly controlled to avoid aberrant protein expression, but the molecular mechanisms that regulate the process are not understood yet. The release of mRNAs occurs on transport RNPs [5], which contain, besides RBPs, also eukaryotic initiation factors (eIFs) and ribosomes [5], [7], [8]. So far it has been established that many RBPs associate to ribosomes, but whether the binding of mRNA-RBP complexes to specific ribosomal proteins is critical to stimulate the post-translatonal modifications of RBPs and, consequently, the release and translation of the associated mRNAs has not been determinated yet.

The role for many ribosomal proteins is mainly structural, whereas for others a double role is emerging: they also participate in the control of specific pathways, as reported by the interaction of some large and small ribosomal proteins with the tumor suppressor p53 [9]. Associated to ribosomes, there are also several proteins whose function is not clear yet. The Receptor Activated C Kinase 1 (RACK1) protein is one example. RACK1 has been isolated as a scaffold protein for the activated PKCβII [10], but several reports have demonstrated its involvement in multiple biochemical pathways [11], [12], [13]. The presence of RACK1 on ribosomes is well documented in mammalian as well as in yeast cells [14], [15], [16]. Moreover by crystallographic studies of Saccharomyces cerevisiae subunit and of Tetrahymena thermophila 40S ribosomal subunit show that RACK1 localizes at head the back of 40S head region and makes direct contact with the ribosomal RNA [17], [18]. RACK1 has been also demonstrated to be recruited in stress granules (SGs) [19]. These structures appear in the cytoplasm in response to stress conditions (heat stress, glucose deprivation, hypoxia) and are constituted by 40S ribosomal subunits, eIFs and many RBPs, such as ZBP1, FMRP and Staufen [20], [21]. Despite the evidence showing RACK1 associated to ribosomes, its possible role in protein synthesis has not been identified. In this study, we show that RACK1 represents a docking site on ribosomes for the β-actin mRNA/ZBP1 complex and that the binding of this complex to RACK1 is critical to the release and translation of β-actin mRNA. This study defines RACK1 as a “ribosome receptor” and provides a new insight into the molecular mechanism for translational control and the release of β-actin mRNA

## Results

### RACK1 colocalizes on transport RNPs *in vivo* and *in vitro*

**Figure 1:**
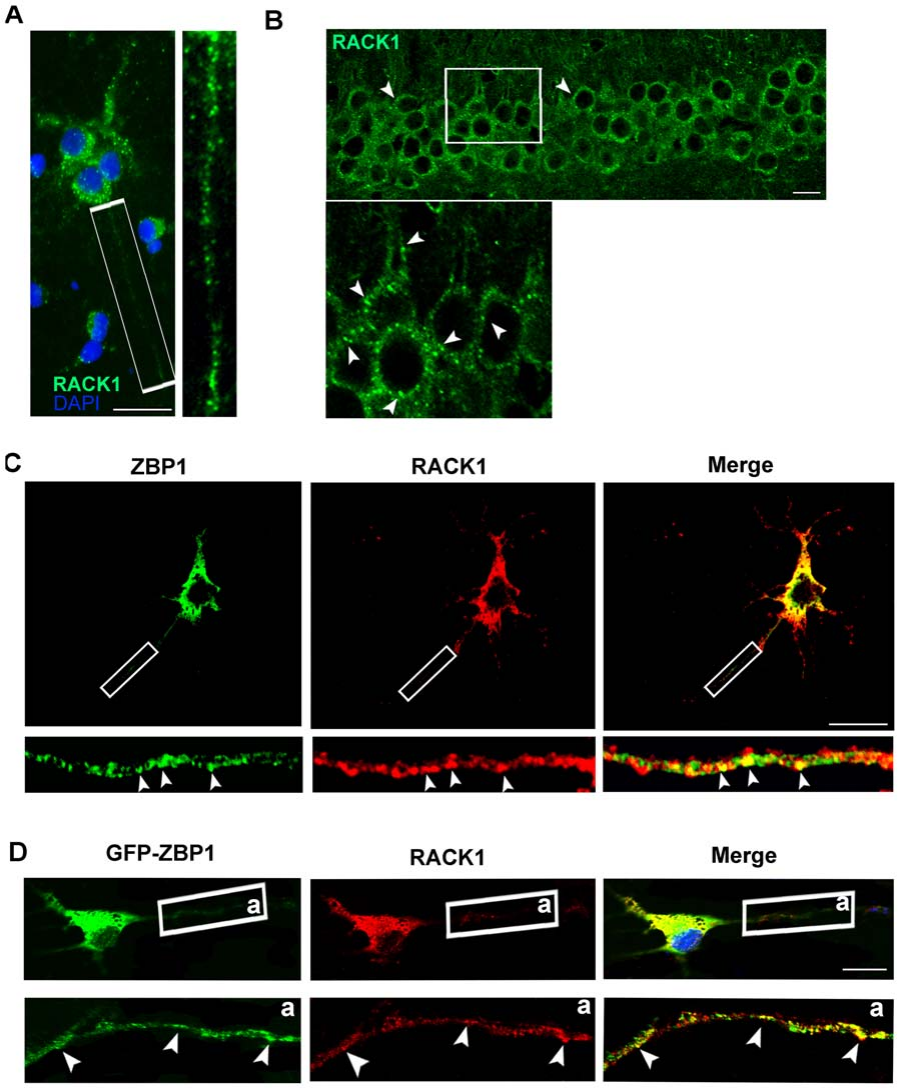
RACK1 localizes on RNA transport granules in cortical neurons. **A**, RACK1 appears in granular forms in rat cortical neurons immunostained with anti-RACK1(*green*) and DAPI (*blue*). Scale bar 20 µm. *Enlarged view* indicates neurite of cortical cells. **B**, RACK1 also appears in granular forms *in vivo*. Hippocampal tissue of adult mouse immunostained with RACK1 (*green*). *Enlarged view* shows the soma of neurons. *Arrows* indicate the granules stained by RACK1 antibody. Scale bar 20 µm. **C**, RACK1 colocalizes with endogenous ZBP1 transport RNPs. Cortical neurons were immunostained with RACK1 (*red*) and ZBP1 (*green*) antibodies. Scale bar 40 µm. *Arrows* in enlarged images indicate granules where RACK1 co-localizes with endogenous ZBP1 along neurites. **D**, RACK1 and GFP-ZBP1 co-localize on transport RNPs of GFP-ZBP1 transfected neurons. Cortical cells were transfected with GFP-ZBP1 cDNA and after 24 hours fixed and processed for anti-RACK1. The *arrows* in e*nlarged view (* ***a*** *)* indicate the granules where GFP-ZBP1 and RACK1 colocalize. Scale bar 20 µm.

RACK1 has been found to be abundantly and widely expressed in almost all areas of the central nervous system. Many studies concerning its scaffold function have been carried out on neuronal tissue [22]. Thus, to investigate its potential role in translational control, we studied RACK1 in neuronal cultures. Previous works showed that RACK1 was distributed in cell bodies and along dendrites of adult tissue mouse [14], [22]. Immunofluorescence studies conducted on cultured embryonic cortical neurons confirmed the sub-cellular localization of RACK1 in soma (Fig. 1A) and along dendrites and axons as shown by colocalization with MAP2 and Tau proteins respectively (Figure S1A and S1B) Moreover, RACK1 appeared in the form of granules in cell body and in neurites (Fig. 1A). To verify whether this granular pattern was also visible *in vivo*, we immunostained slices of adult mouse hippocampus by anti-RACK1 antibody. In adult tissue, RACK1 showed a granular distribution identical to that in cortical neurons (Fig. 1B). This demonstrated that RACK1 distributed in granular form both *in vitr*o and *in vivo*.

To verify whether the distribution of RACK1 was affected by neuronal activity, cortical neurons were depolarized with 50 mM KCl for 15 min and, next, immunostained by anti-RACK1 antibody. KCl significantly increased the number of granules labeled by RACK1, indicating the sensitive of its distribution to membrane depolarization (Figure S2). Many RBPs and ribosomal proteins of transport RNPs show a pattern similar to that observed by RACK1. Moreover, they redistribute as RACK1 in response to KCl stimuli [23]. Thus, we verified whether RACK1 might be part of transport RNPs. A co-immunostaining analysis in primary cortical neurons with anti-RACK1 and anti-ZBP1 antibodies, a specific marker for transport RNPs [24] revealed an extensive co-localization of RACK1 and ZBP1 proteins in the soma. Along neurites some particles stained by RACK1 were also positive for ZBP1, while others are located next to each other (Fig. 1C). To further confirm the localization of RACK1 on transport RNPs, the granular pattern of RACK1 was investigated in cortical neurons transfected with GFP-ZBP1, which is known to be recruited in RNPs [5]. We found that, in transfected neurons, the particles stained by RACK1 strongly colocalized with those of GFP-ZBP1 in soma and in neurites (Fig. 1D). Taken together, these results confirmed the localization of RACK1 on transport RNPs of cortical neurons.

### RACK1 interacts with ZBP1 through its Src phosphorylation and binding site (Y246)

**Figure 2:**
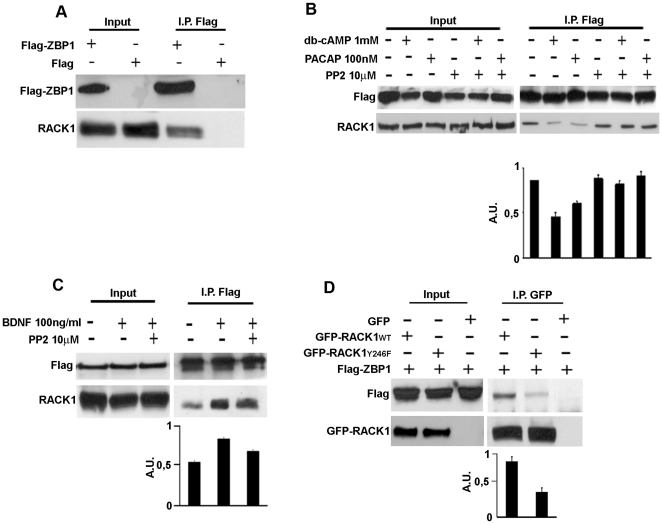
Src kinase regulates the RACK1/ZBP1 complex and the Y246 of RACK1 is critical for the binding with ZBP1. **A**, Endogenous RACK1 interacts with Flag-ZBP1 in an anti-Flag immunoprecipitation assay from total lysate of neuroblastoma Flag-ZBP1-transfected cells. Western blotting for endogenous RACK1 and Flag-ZBP1 on proteins eluted from anti-Flag immunoprecipitation assay. Flag transfected cells were used as negative control. Input represents 5% of total lysate. **B** and **C**, Src activity regulates the RACK1-ZBP1 complex formation. **B**, db-cAMP or PACAP treatments of Flag-ZBP1 transfected cells reduced the binding between RACK1 and Flag-ZBP1, whereas PP2 restored the binding, as in untreated cells. **C** BDNF treatments increased the binding of Flag-ZBP1 to RACK1 in Flag-ZBP1 transfected cells. Src inhibition by Src inhibitor PP2 reduced the RACK1-ZBP1 complex stimulated by BDNF. The density value of immnoprecipitated RACK1 is normalized to that of immunoprecipitated Flag-ZBP1 and summarized in both graphics. Data are graphed as means plus S.D. **D**, The Src binding and phosphorylation site (Y246) of RACK1 is critical for complex formation. Flag-ZBP1 protein co-immunoprecipitated with GFP-RACK1_wt_ and GFP-RACK1_Y246F_ in immunopreciptation assays using anti-GFP antibody, but in the presence of GFP-RACK1_Y246F_ the binding was reduced. The figures are representative of three independent experiments. The density value of co-immnoprecipitated Flag-ZBP1 is normalized to that of immnoprecipitated GFP-RACK1_wt_ or GFP-RACK1_Y246F_ and summarized in both graphic. Data are graphed as means plus S.D.

The strong colocalization of RACK1 with ZBP1 on transport RNPs prompted us to investigate whether RACK1 might interact with ZBP1. To this aim, human SH-SY5Y neuroblastoma cells, expressing low amount of ZBP1 (data not shown), were transfected with Flag-ZBP1 cDNAs and a co-immunoprecipitation assay was performed from total lysates. As revelead by western blotting analysis on eluted protein from Flag and Flag-ZBP1 immunoprecipitation, RACK1 associated to Flag-ZBP1 (Fig. 2A), indicating a specific interaction with ZBP1.

Next, we asked how the interaction of RACK1 with Flag-ZBP1 might be regulated. It has been reportd that RACK1 is phosphorylated by Src kinase and that this phosphorylation is critical for the binding of Src to RACK1. Moreover, we previously reported that ZBP1 protein is a substrate of Src tyrosine kinase [6], [13]. Thus, we postulated that Src kinase might control the RACK1-ZBP1 interaction. We studied the binding of RACK1 to ZBP1 in response to treatments known to activate Src, such as dibutyl-cAMP (db-cAMP), Pituitary Adenylate cyclase-activating polypeptide (PACAP) or BDNF [25], [26] in presence of the specific Src inhibitor, PP2. In the db-cAMP or PACAP stimulated cells, we found that the interaction of RACK1 to Flag-ZBP1 was reduced, while PP2 restored the complex at levels comparable to control cells (Fig. 2C). On the contrary, BDNF increased the binding of RACK1 to Flag-ZBP1, while PP2 reduced the association between the proteins (Fig. 2D). These results indicated that Src may associate/dissociate the Flag-ZBP1/RACK1 complex, depending on which extracellular signal stimulates its kinase activity.

To further investigate how Src may regulate the interaction of RACK1 with ZBP1, we co-expressed, in SH-SY5Y cells, Flag-ZBP1 with GFP-RACK1_wt_, which is reported to inhibit the Src kinase activity [11], [13], or with GFP-RACK1 mutated in Src phosphorylation and binding site (tyrosine 246, Y246 changed in phenyalanine, Y246F), which on the contrary does not inhibit Src activity [11], [13]. If Src controls the association/dissociation of RACK1-ZBP1 complex, the overexpression of GFP-RACK1_wt_ should inhibit the association or the dissociation of complex after Src activation. While the overexpression of mutated RACK1 should stimulate it. As shown by immunoblotting for Flag-ZBP1 on proteins eluted in GFP-immunoprecipitation, GFP-RACK1_wt_ binds Flag-ZBP1 in untreated control conditions (Fig. 2D). Surprisingly, in GFP-RACK1_Y246F_/Flag-ZBP1 co-expressing cells, GFP-RACK1_Y246F_ associated with Flag-ZBP1 less than GFP-RACK1_wt_, suggesting that RACK1 and ZBP1 interacted through the Src binding site of RACK1.

### RACK1_Y246F_ inhibits recruitment of ZBP1 on ribosomes and reduces the release and translation of β-actin mRNA

**Figure 3:**
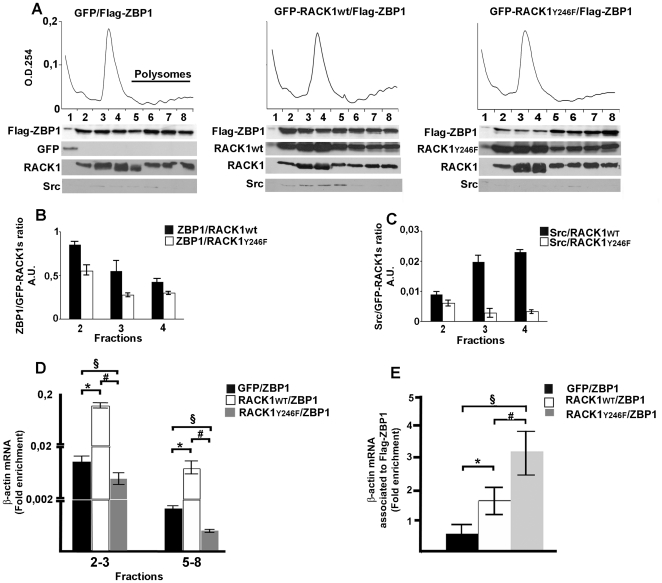
RACK1_Y246F_ impairs the binding of ZBP1 and Src kinase to ribosomes and reduces the release and translation of β-actin mRNA. **A**, ZBP1 and Src are less abundant in ribosomal profile fractions collected from stable GFP-RACK1_Y246F_ overexpressing cells, transfected with Flag-ZBP1 cDNA. The histograms in **B** and in **C** summarize the ratio of density values between Flag-ZBP1 **B** or Src **C** and GFP-RACK1_wt_ or GFP-RACK1_Y246F_ immunoblots in 2–4 ribosomal fractions. **D**, The level of β-actin mRNA, measured by qRT-PCR, on RNA isolated from non translating fractions (fractions 2–3 of ribosomal profile reported in **A**) and from polysomal fractions (fractions 5–8) was decreased in GFP-RACK1_Y246F_/Flag-ZBP1 cells, as compared to that of GFP-RACK1_wt_/Flag-ZBP1 expressing cells. **E**, GFP-RACK1_Y246F_ induced an increase of β-actin mRNA associated to Flag-ZBP1, as measured by qRT-PCR on RNA isolated from Flag-ZBP1 immunoprecipitation of GFP-RACK1_Y246F_/Flag-ZBP1 cells. In **D** and **E**, the values were normalized to those of 18S rRNA and the data are graphed as mean ± S.D.* = p<0,05, # and § = p<0,01 second *t-test* student. In A and D the transfection efficiency was normalized with respect to the amount of Actin protein (Figure S3). In E, the transfection efficiency was normalized with respect to immunoprecipitated Flag-ZBP1 proteins as in Figure 2B.

It has been described that the Src-binding site of RACK1 remains exposed when it is associated with the 40S subunit [17], [18]. Moreover, Src removes the translational repression on β-actin mRNA by phosphorylating ZBP1 at Tyr396 [6]. The results presented so far establish that the Src phosphorylation and binding site Y246 of RACK1 is critical for the association of RACK1 to ZBP1. Therefore, we asked whether, through this site, RACK1 may represent a scaffold protein for β-actin mRNA/ZBP1 complex on ribosomes. To verify this hypothesis, we investigated the localization of Flag-ZBP1 on ribosome in GFP-RACK1_Y246F_ overexpressing cells. Ribosomal profiles were conducted on neuroblastoma cells co-expressing Flag-ZBP1 with GFP, GFP-RACK1_wt_ or with GFP-RACK1_Y246F_ cDNAs. Immunoblotting with Flag, GFP and RACK1 antibodies, assessed on collected fractions, revealed that Flag-ZBP1, GFP-RACK1_WT_ and GFP-RACK1_Y246F_ co-sedimented in free ribosomes and polysomes as endogenous RACK1. As expected, the presence of GFP-RACK1_Y246F_ on ribosomes greatly reduced the amount of Flag-ZBP1 on fractions corresponding to 40S and 60S/80S subunits (fractions 2–4 in Fig. 3A, and graphic in 3B). Since the Y246F mutation did not affect GFP-RACK1_Y246F_ nor endogenous RACK1 levels in ribosomal fractions (Fig. 3A), the low amount of Flag-ZBP1 on ribosome subunits was be dependent on the decrease of its binding to GFP-RACK1_Y246F_.

Given that Src may regulate the RACK1/Flag-ZBP1 complex formation, we evaluated whether GFP-RACK1_Y246F_ expression also impaired the localization of Src on ribosomes. An active role of Src in translational control has been documented [27], [28], but its direct association to translational machinery has not been demonstrated yet. Ribosomal profiles of SH-SY5Y cells, showed that Src co-sedimented in the same fractions containing eIF4E, an eukaryotic factor of 48S complex [29] (Figure S4A). Moreover, the binding of Src to the translational machinery was confirmed by its co-purification with RACK1 and polyA-binding protein (PABP), in an oligo-dT pull down assay as performed in Text S1 (Figure S4B). In SH-SY5Y co-expressing Flag-ZBP1 and GFP-RACK1_Y246F_ cells, the localization of Src on ribosomes was reduced, indicating that the GFP-RACK1_Y246F_ on ribosomes also impaired the binding of Src to 40S/60S and to 80S monosomes (Fig. 3A and graphic in 3C).

The binding of the β-actin mRNA to ZBP1 [5], [6] and the impairment of association of Flag-ZBP1 to GFP-RACK1_Y246F_ at the ribosome level led us to investigate whether the amount of β-actin mRNA bound to ribosomes and its translation were also affected. To this aim, the level of β-actin mRNA was measured by qRT-PCR on total RNA purified from translationally inactive (fractions 2–3 of ribosomal profile in Fig. 3A) and translationally active fractions (polysomal fractions 5–8). The assay was performed in Flag-ZBP1 overexpressing cells transfected with GFP, GFP-RACK1_wt_ or GFP-RACK1_Y246F_ cDNAs. In cells co-expressing Flag-ZBP1 and GFP-RACK1_wt_ the amount of β-actin mRNA was increased in both translationally inactive and in polysomal fractions (Fig. 3D). The GFP-RACK1_Y246F_ overexpression greatly reduced the level in both fractions compared to those of GFP-RACK1_wt_, indicating a low rate of translation. Moreover, the total β-actin expression mRNA was not affected by the GFP-RACK1_WT_ and GFP-RACK1_Y246F_ overexpression (Figure S5). These results demonstrated that the decrease of β-actin mRNA on ribosomes and of its translation was not dependent on transcriptional control, but on the decrement of GFP-RACK1_Y246F_/Flag-ZBP1 complex on ribosomes

The release of β-actin mRNA from ZBP1 is an essential step for its translation. Therefore, we asked whether GFP-RACK1_Y246F_ might also affect the release of β-actin mRNA from ZBP1. We quantified the amount of β-actin mRNA bound to Flag-ZBP1 by qRT-PCR assay on RNA purified by Flag-ZBP1 immunoprecipitation. We conducted the assay on Flag-ZBP1 overexpressing cells transfected with GFP, GFP-RACK1_wt_ and GFP-RACK1_Y246F_ cDNAs. The level of β-actin mRNA associated to Flag-ZBP1 in cells co-transfected with GFP-RACK1_Y246F_ and Flag-ZBP1 cDNAs was greater than that in cells co-transfected with Flag-ZBP1 and GFP or GFP-RACK1_wt_ cDNAs (Fig. 3E). This suggested that the β-actin mRNA accumulated on Flag-ZBP1 protein and its release from Flag-ZBP1 was impaired.

Taken together, these results showed that GFP-RACK1_Y246F_, affecting the binding of ZBP1 and of Src to ribosomes, reduces the release of β-actin mRNA from Flag-ZBP1 and, consequently, its translation

### The translation of β-actin mRNA in the growth cone is reduced by RACK1_Y246F_

**Figure 4:**
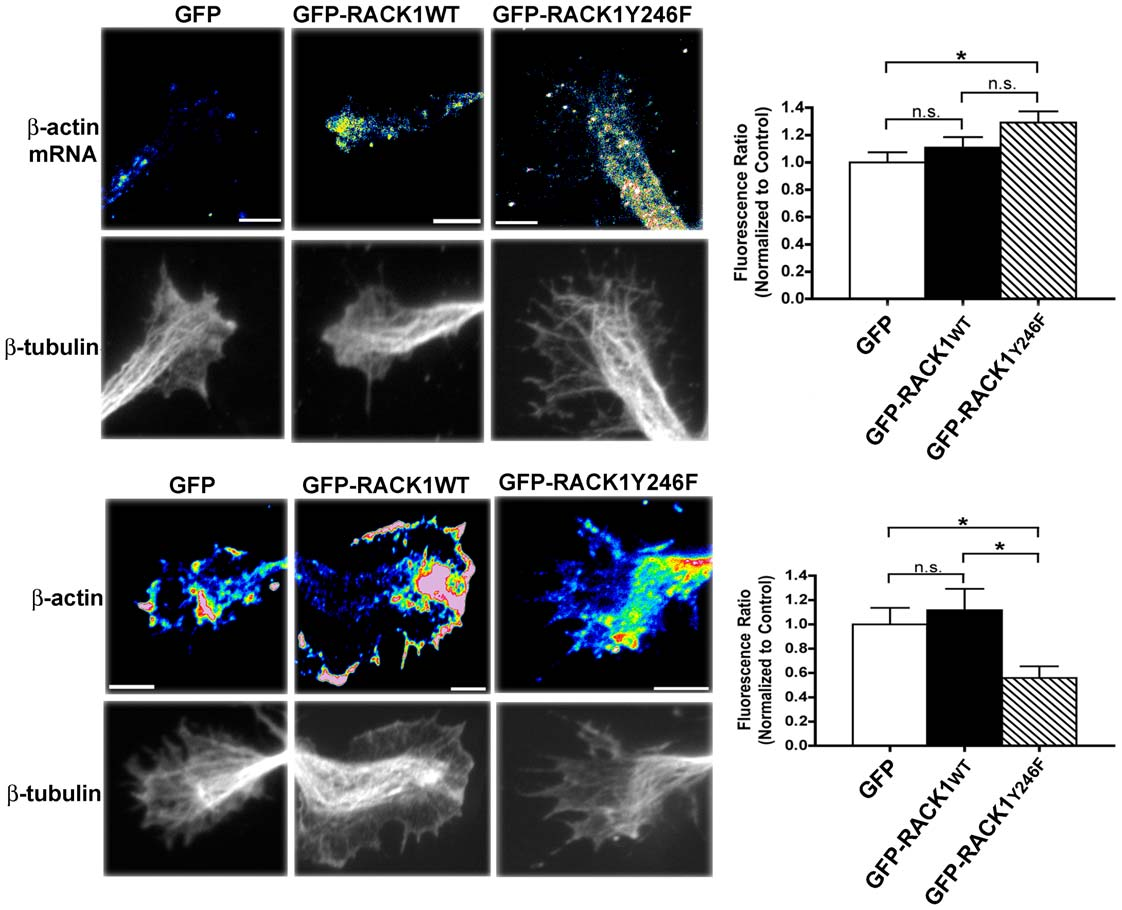
RACK1_Y246F_ induces accumulation of β-actin mRNA in growth cones of cortical cells and reduces its translation. **A**, Growth cones of cortical cells co-transfected with Flag-ZBP1_wt_ and GFP-RACK1_Y246F_ show increased levels of β-actin mRNA as measured by Q-FISH. The method to quantify the signal is reported in supplemental information and the values are summarized in the histogram in the left. **B**, The translation of β-actin protein in growth cones of cortical neurons co-transfected with Flag-ZBP1_wt_ and GFP-RACK1_Y246F_ was reduced, as indicated by in Q-IF. All data are reported as mean ± s.e.m. Significance was set as p≤0.05. When significance was adjusted, it is referred to as alpha. β-tubulin immunofluorescence is shown as a marker for growth cone morphology. Scale bar 10 µm.

The release and the translational control of β-actin mRNA by Src phosphorylation of ZBP1 plays a critical role in growth cones [6]. The results in neuroblastoma cells indicate that the decrease of the binding of β-actin mRNA/ZBP1 complex to GFP-RACK1_Y246F_ on ribosomes reduces the release of β-actin mRNA from ZBP1 and, consequently, its translation. Thus, we asked whether GFP-RACK1_Y246F_ overexpression also affected the release and translation of β-actin in growth cones. To this aim, cortical neurons were co-transfected with Flag-ZBP1 and GFP-RACK1_wt_ or GFP-RACK1_Y246F_ cDNAs. The growth cones were analyzed by FISH, to evaluate the amount of β-actin-mRNA, and by immunofluorescence, to detect the level of endogenous β-actin protein (as reported in Materials and Methods and in Text S1). Quantitative fluorescence analysis showed that FISH signals (Fig. 4A) in growth cones of neurons co-expressing GFP-RACK1_wt_ and Flag-ZBP1 were similar to those of neurons co-expressing GFP and Flag-ZBP1 (control neurons). In contrast, a significant increase of the mRNA fluorescence intensity, with respect to control neurons, was observed in growth cones of neurons co-expressing GFP-RACK1_Y246F_ and Flag-ZBP1. This showed that there was an accumulation of β-actin mRNA in the growth cones of neurons expressing GFP-RACK1_Y246F_, probably as a consequence of an impaired release from Flag-ZBP1, as observed neuroblastoma cells (Fig. 3E). The quantification of immunofluorescence experiments (Fig. 4B) confirmed, at the protein level, the results of FISH studies. In fact, a strong decrease of fluorescence signal for endogenous β-actin protein was observed in growth cones of neurons co-transfected with GFP-RACK1_Y246F_ and Flag-ZBP1 cDNAs. This indicated a reduction of endogenous β-actin protein. which might affect the incorporation of β-actin protein into actin filament and, consequently impair the growth cones. Thus, the results from growth cones of cortical cells, as well as in neuroblastoma cells indicated that, the release from Flag-ZBP1 and the translation of β-actin mRNA were inhibited by GFP-RACK1_Y246F_.over-expression.

### The overexpression of GFP-RACK1 stimulates the dendritic branching out in cortical neurons

**Figure 5:**
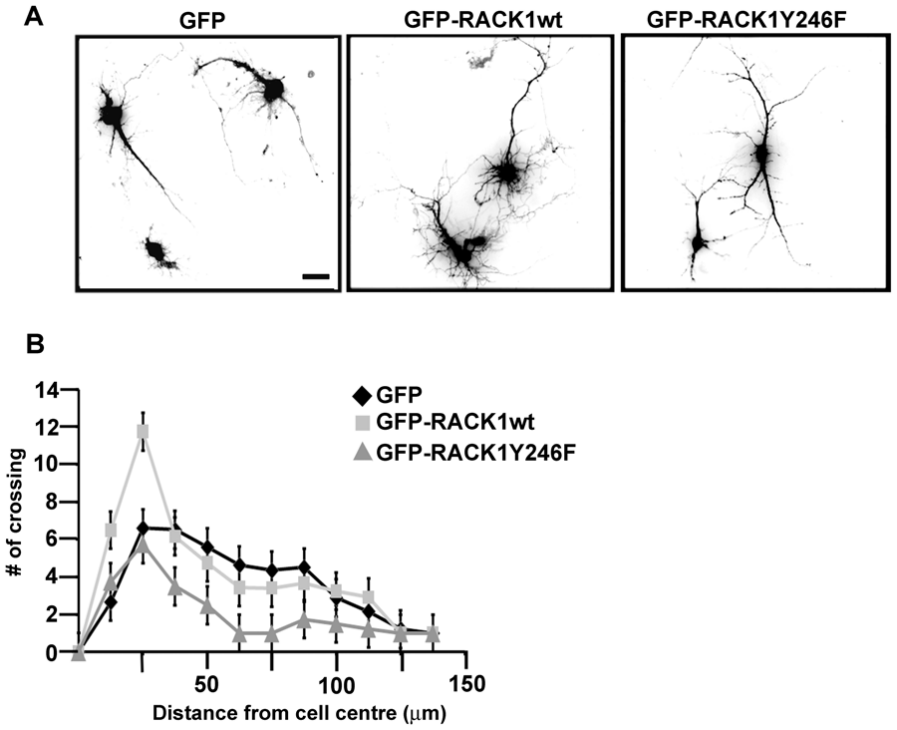
The dendritic branching out induced by GFP-RACK1wt. **A**, Immunofluorescence for GFP in cortical neurons transfected with GFP, or GFP-RACK1_wt_ or GFP-RACK1_Y246F_. GFP-RACK1_Y246F_ reduced the dendritic arbors indeced by GFP-RACK1_wt_ overxpression Scale bar 20 µm **B**, Graphic reporting the values of dendritic branching seen in **A**. The values were measured as means of the number of neurite intersections measured by Sholl analysis. Data are graphed as mean ± S.D.

The results obtained in growth cones prompted us to investigate the overall morphological effects induced by RACK1_Y246F_ in neurons. The dendritic morphology of cortical neurons transfected with either GFP-RACK1_wt_ or GFP-RACK1_Y246F_, revealed by anti-GFP immunofluorescence (Fig. 5A), was very striking. Quantitative analysis of dendritic morphology measured by Sholl analysis (as reported in Text S1) showed greater neurite branching in GFP-RACK1_wt_ expressing cells than in GFP and GFP-RACK1_Y246F_ transfected neurons. There was a higher number of thin dendritic arborizations in GFP-RACK1_wt_ expressing cells. In some cells, the arborizations departed directly from the soma and in others from the principal dendritic processing (Fig. 5A and graphic in B). These results demonstrate that, although the Src site on RACK1 protein is fundamental to regulate the dendritic outgrowth by controlling the β-actin mRNA translation, RACK1 may contribute to the dendritic arbors through regulation of other pathways.

## Discussion

In this study we show that RACK1 functions as a scaffold protein on ribosomes for the β-actin mRNA/ZBP1 complex. We found that the binding of RACK1 to ZBP1 on ribosomes is critical for the release of β-actin mRNA from ZBP1 and, consequently, for its translation. The presence of RACK1 on ribosomes has been reported by several studies [14], [15]. Despite the protein sequence of RACK1 is lacking of RNA-binding domain, the disruption of yeast RACK1 ortholog's (Asc1p) position at 40S ribosomal subunit results in failure of the mRNA binding protein Scp160 to associate with ribosomes [16]. Recently, it has been reported that RACK1 can contribute to the association of miRNA complex to the translating ribosomes [30], [31]. Recent papers on the three-dimensional structure of eukaryotic 40S and 80S demonstrate that RACK1 could be available for simultaneous binding to ribosomes and Src and/or PKC [17], [18]. Indeed, in the structure of RACK1 bound to 40S ribosome subunit, the Src binding and the PKC binding domains of RACK1 are exposed. Thus, in this context, it is plausible to identify the function of RACK1 on ribosomes through the interaction with Src substrates such as ZBP1.

**Figure 6:**
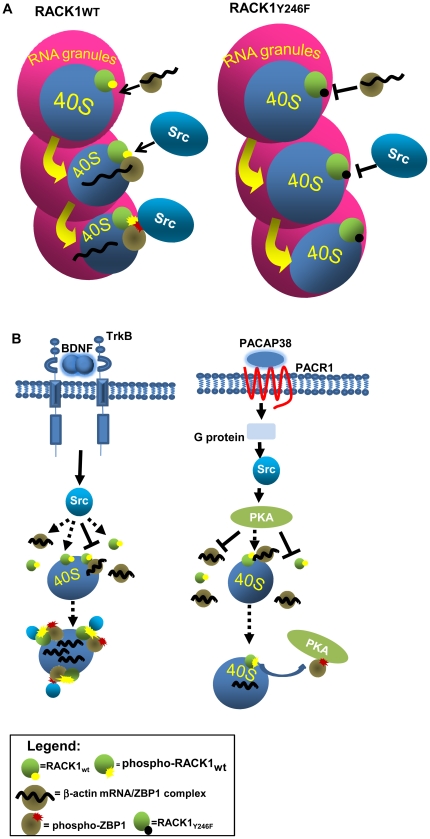
Molecular mechanism of RACK1/ZBP1 complex regulating the release and translation of β-actin mRNA. A, Schematic figure showing the interaction of RACK1 with the β-actin mRNA/ZBP1 complex through its Src binding site (Y246) on 40S ribosome subunit of RNPs (*left*). In presence of the mutation Y246F (*right*), RACK1 on ribosomes fails to recruit the β-actin mRNA/ZBP1 complex and Src. This impairment blocks the release of β-actin mRNA from ZBP1 and its translation. B, Src can be activated by BDNF or by PACAP treatments. In BDNF stimulation, Src, activated through TrkB, may directly bind, and phosphorylate, both free or ribosomal bound RACK1 and free β-actin mRNA/ZBP1 complex. Next, RACK1 and β-Actin mRNA/ZBP1 associate on ribosomes and the β-actin mRNA is released to be translated. Instead, PACAP-activated Src stimulates PKA kinase which in turn may induce the dissociation of the β-actin mRNA/ZBP1-RACK1 complex by phosphorylating ZBP1.

Here, we demonstrate that RACK1 binds the β-actin mRNA/ZBP1 complex through the Src phosphorylation and binding site Y246. It is well established that phosphorylation of this site by Src is a prerequisite for the binding of Src to RACK1 and for kinase inhibition [11], [32]. Our results suggest that, in presence of ZBP1, ribosomal RACK1 preferentially binds ZBP1 at the Y246 site. This interpretation is confirmed by release of β-actin mRNA from Flag-ZBP1 in cells and in growth cones expressing GFP-RACK1_wt_. In fact, the expression of GFP-RACK1_wt_, demonstrated to inhibit Src kinase activity [11], [32], increases the release of β-actin mRNA from Flag-ZBP1 and, consequently, its translation, compared to those of GFP-RACK1_Y246_. This indicates that Src can phosphorylate ZBP1 to remove the repression on β-actin mRNA, and demonstrates that Src is active and dissociated from GFP-RACK1_wt_. This model is fully in agreement with ZBP1 phosphorylation by Src to remove the translational repression at β-actin mRNA in specific sites of neurons [33]. Indeed, the localization of endogenous RACK1 and ZBP1 on transport RNPs (Fig. 1c and D) may repress the translation of β-actin mRNA during transport to specific sites. At local destinations, activated Src removes the translational repression by phosphorylating ZBP1. The role of Src in this molecular mechanism may be distinct depending on which extracellular stimuli induce its activity. The BDNF-activated Src may directly stimulate the association of ZBP1 to RACK1, through a direct binding to both proteins, whereas PACAP38-activated Src may indirectly induce the dissociation of ZBP1-RACK1 complex, through the activation of PKA (Fig. 6B). This latter hypothesis is supported by findings that PKA is activated by Src, following PACAP signalling [26] and by the presence of phosphorylation consensus site for PKA on ZBP1 [34]. Further studies will be essential to establish whether the regulation of ZBP1/RACK1 complex by Src kinase is a further mechanism for translational control of other mRNAs bound to ZBP1.

The translational control by Src activity has been well documented. It has been reported that Src acts on eIFs regulating the mTOR kinase [27], [28], [35], [36]. However, in this mechanism, Src controls the translation of all cap-mRNAs and of the overall protein synthesis. Instead, in the translation of β-actin mRNA, Src, by phosphorylating ZBP1, directly acts on the translational control of specific mRNAs. Our findings show that Src localizes on ribosomes, suggesting that it may act at ribosomal level to control the translation of specific mRNAs. In the absence of ZBP1, Src may bind to ribosomes through the binding and phosphorylation site of RACK1, in agreement with proposed model in crystallographic studies on ribosomal RACK1 [17], [18]. In this case, we speculate that, in correlation with the ability of RACK1 to inhibit the Src kinase [13], the binding of Src to RACK1 on ribosome may be necessary to avoid aberrant phoshorylations of some components of translational machinery. The presence of Src on ribosomes opens the possibility to consider new potential Src ribosomal substrates in the regulation of translation.

Several evidences in neurons have shown that RACK1 is involved in multiple neuronal functions by the association to a variety of proteins, such as kinases, phosphatases and critical membrane receptors [23]. In neurons, RACK1 has been localized in cell bodies and along dendrites [14], [22]. Here, we show that both *in vitro* and *in vivo* RACK1 appeared in granular forms corresponding to transport RNAs. Our study on RACK1 overexpression in cortical neurons indicate that it may regulate the dendritic branching out. We propose that the scaffold properties of RACK1 may favor the binding on ribosomes, besides of ZBP1, also of other protein regulating this neuronal processing. This is corroborated, as seen above, by structural studies which show that RACK1 bound to 40S subunit might bind not only Src kinase, but also PKC kinase. Although it is possibile that RACK1 may regulate the branching out through the interaction with other proteins, the results observed on overexpression of RACK1 are in agreement with the critical role of ZBP1 in dendritic arborization development in hippocampal neurons [37]. The identification of which other specific mRNA/RBPs complexes are associated to RACK1 and which other mRNAs are translational controlled by RACK1 will help to elucidate the function of RACK1 in neuronal cells.

## Materials and Methods

### Cell Culture, transfections and stable clones

Human neuroblastoma SH-SY5Y cells, obtained from American Type Culture Collection (ATCC, Rockville, MD) were cultured in DMEM/F12 medium containing 10% of FBS and antibiotics (50 U/ml penicillin and 50 µg/ml streptomycin) at 37°C in 5% CO_2_/95% air. Cortical and hippocampal neuron cultures were prepared from Sprague-Dawley E18 embryos, as previously described [6], [38]. For transfections, SH-SY5Y cells were transfected with Flag, Flag-ZBP1, pEGFP, GFP-RACK1_wt_ and GFP-RACK1_Y246F_ cDNAs using the manufacturer's protocol of Lipofectamine 2000 (Invitrogen). Stable SH-SY5Y clone cells for pEGFP, GFP-RACK1_wt_ and GFP-RACK1_Y246F_ were produced by selecting transfected cells resistant at 450 µg/ml G418 (Gentamycin, Sigma). Neurons were transfected with the rat Amaxa nucleofection kit (Lonza) or Lipofectamine 2000 (Invitrogen). In Amaxa nucleofection, 5×10^6^ neurons were transfected with 1.5 µg of Flag-ZBP1 and 1.5 µg of a GFP plasmid (pEGFP, GFP-RACK1_wt_, GFP-RACK1_Y246F_, GFP-ZBP1). For Lipofectamine 2000, neurons were processes as indicated by the manufacturer's protocol, using 1.5 µg of Flag-ZBP1 and 1.5 µg of GFP plasmids.

For pharmacological treatments, SH-SY5Y Flag-ZBP1 transfected cells were starved 24 hrs and then treated with 100 ng/ml BDNF (Alomone),1 mM dibutyryl-cAMP. (db-cAMP, Sigma) or 100 nM Pituitary adenylate cyclase-activating polypeptide (PACAP, Sigma) for 30 min at 37°C. Where required, 10 µM Src inhibitor PP2 (Calbiochem) or vehicle were added to the medium for 30 min at 37°C. For cortical embryonic cells, 50 mM KCl was added to Neurobasal B27 15 min before fixation.

### Fluorescent In Situ Hybridization (FISH) and Immunofluorescence (IF)

Q-FISH was performed as previously described in text S1 and in [6]. One digoxigenin-labeled oligonucleotide probe was used to detect rat β-actin mRNA. The sequence of this probe was: 5′-TGAGGAAAGTAGGGTTGATGAGGCCAGCTTGGCCAGGTGTCAGGGAGATACCTTC-3′ As controls, neurons were hybridized with a DIG-labeled scrambled oligonucleotide probe

### Immunofluorescence (IF) experiments

IF experiments were performed essentially as previously described in Text S1 and in [6], and the following primary antibodies were used: mouse anti-RACK1 (BD biosciences, 1∶100), polyclonal anti-ZBP1 mouse [39] anti-tubulin (E7, Developmental Studies Hybridoma Bank; 1∶1500), rabbit anti-tubulin (Sigma; 1∶1000), mouse anti-β actin (AC15, Abcam; 1∶1500), rabbit anti-Flag (Sigma; 1∶1000), polyclonal anti-GFP (Text S1, 1∶2000).

### Immunohistochemistry

Three-months-old mice were anesthetized with an excess of 2,2,2-tribromethanol (400 mg/kg) and intracardially perfused with a 4% solution of paraformaldehyde in PBS. Brains were processed for immunohistochemistry to detect RACK1 using the primary antibody seen above. Immunohistochemistry was performed according to the protocols previously described [40].

All experiments were conducted according to national and international laws for laboratory animal welfare and experimentation (EEC Council directive 86/609, OJ L 358, 12 December 1987. Experimentation was approved by Italian Department of health (approval n. 9/2006).

### Ribosome profiles, immunoprecipitations and western blotting

For ribosome profiles, wild type or transfected SH-SY5Y cells were lysed in polysomal buffer (10 mM Tris-HCl, 50 mM KCl, 10 mM MgCl_2_ and 0,5% NP-40). MgCl_2_ was substituted with 10 mM EDTA, for ribosome profile in the presence of EDTA. Total lysate was clarified by centrifugation at 14,000 r.p.m. for 5 min at 4°C and the supernatanat was loaded on a continuous sucrose gradient 15–50% in 10 mM Tris-HCl, 50 mM KCl, 10 mM MgCl_2_ or 10 mM EDTA. After ultracentrifugation (Beckman) at 37,000 rpm for 2 hrs at 4°C, sucrose gradient was collected in fractions and the profile was obtained by total RNA analysis at 254 nm in Bio-radBiologic LP Half amount of collected fractions was used to precipitate proteins with 10% trichloroacetic acid (TCA) and the other half used to isolate total RNA.

Immunoprecipitations were conducted in polysomal buffer and SH-Y5Y transfected cells were lysate and clarified as seen for ribosome profiles. Next, the supernatant was incubated overnight either with polyclonal-GFP, produced as in Text S1, (1∶100) or 30 µl mouse anti-Flag M2 (Sigma-Aldrich) at 4°C. For GFP immunoprecipitation, pre-equilibrated 30 µl of protein G and 30 µl of protein A sepharose resin (GE healthcare) were added and incubated for 1 hour at 4°C. The resins were extensively washed with polysomal buffer and total RNA or proteins were eluted for RNA isolation or for western blotting.

For western blotting, proteins were loaded on SDS-PAGE 10% and transferred on PVDF (Millipore) membrane and the following primary antibodies were used: mouse anti-RACK1 (1∶2000), mouse anti-ZBP1 (Huttelmaier 2005, 1∶1000), polyclonal anti-GFP (1∶1000), mouse anti-Actin (Sigma, 1∶1000), polyclonal anti-Src ,polyclonal anti-eIF4E, polyclonal anti-PABP (alls from Cell signalling, 1∶1000) and mouse anti-GAP43 (1∶2000), polyclonal anti-p27^BBP^/eIF6 (kindly provided by prof. Biffo Stefano, 1∶1000). Secondary HRP-coniugated anti-mouse or anti-rabbit antibodies and ECL reagent (GE healthcare) were used. For RACK1, mouse HRP-coniugated anti-IgM was used.

### RNA isolation and qRT-PCR

Total RNA was purified from immunoprecipitates and ribosomal fractions with TriReagent (Invitrogen) according to manufacturer's protocol. The purified RNA was used for qRT-PCR. The first strand cDNA template was synthesized from 500 ng of total RNA using random primers and Superscript III reverse transcriptase (Invitrogen, USA). All reactions were performed with SYBR Green PCR Master Mix (BioRad) and carried out in the iCycler (BioRad). Primers for Quantitative PCR (QTR-PCR) analysis were designed with the assistance of Universal Probe Library Software (Roche Applied Science). The following primers were selected to amplify: Homo sapiens β-actin, (ACTB) forward 5′-TCCCTGGAGAAGAGCTACG-3′ and reverse 5′-GTAGTTTCGTGGATGCCAC; Homo sapiens RNA, 18S (ribosomal 1 forward) 5′-AGGGCAGGGACTTAATCAACGC-3′ and reverse 5′-GTTGGTGGAGCGATTTGTCTGG-3. Relative change of mRNA amount was calculated based DCt method, as described in [41].

## Supporting Information

Figure S1 **RACK1 along dendrites and axons of embryonic cortical cells.** Co-immunostaining of MAP2 (A, *green*) or Tau (B, *green*) and RACK1 (*red*) indicated the localization of RACK1 in dendrites and axons. Scale bar 20 µm.
(TIF)

Figure S2 **Neuronal activity reorganizes the distribution of RACK1.** 50 mM KCl for 15 min increases the number of granules stained by RACK1. In the graphic are summarized the results observed in immunofluorescence *in right* Scale bar 20 µM.
(TIF)

Figure S3 **Transfection efficiency.** Western blotting for GFP, GFP-RACK1, Flag-ZBP1, RACK1, Src and Actin on total lysate from SH-SY5Y cells co-expressing Flag-ZBP1 and GFP, GFP-RACK1_wt_ or GFP-RACK1_Y246F_ protein.
(TIF)

Figure S4 **Src is part of the translational machinery.** A, Src, as well as eIF4E and RACK1, localized in fractions at the top of the gradient, where 40S accumulated in the EDTA sucrose gradient from SH-SY5Y cells. B, Oligo-dT assay in cortical cells which specifically purifies proteins associated to mRNAs such as RNA binding proteins, 40S, 80S, polyribosomes and translational factors (ref 1 in Text S1). Src was specifically isolated as RACK1 and PABP proteins. p27^BBP^/eIF6, which binds only 60S ribosomal subunit and does not bind mRNAs, was not purified, indicating that the assay was specific for proteins associated to mRNAs. The figure shows the western blots on three independent experiments.
(TIF)

Figure S5 **The β-actin mRNA expression in GFP-RACK1_wt_ and GFP-RACK1_Y246F_ overexpressing cells.** Total β-actin mRNA, measured by qRT-PCR and normalized to 18S rRNA, is not affected by overexepression of GFP-RACK1_wt_ and GFP-RACK1_Y246F_.
(TIF)

Text S1 (DOC)
