# Supplementary figures and images for: RACK1 Is a Ribosome Scaffold Protein for β-actin mRNA/ZBP1 Complex
Source: PLoS One. 2012 Apr 16;7(4):e35034. doi: 10.1371/journal.pone.0035034 (PMC3327689; doi:10.1371/journal.pone.0035034)

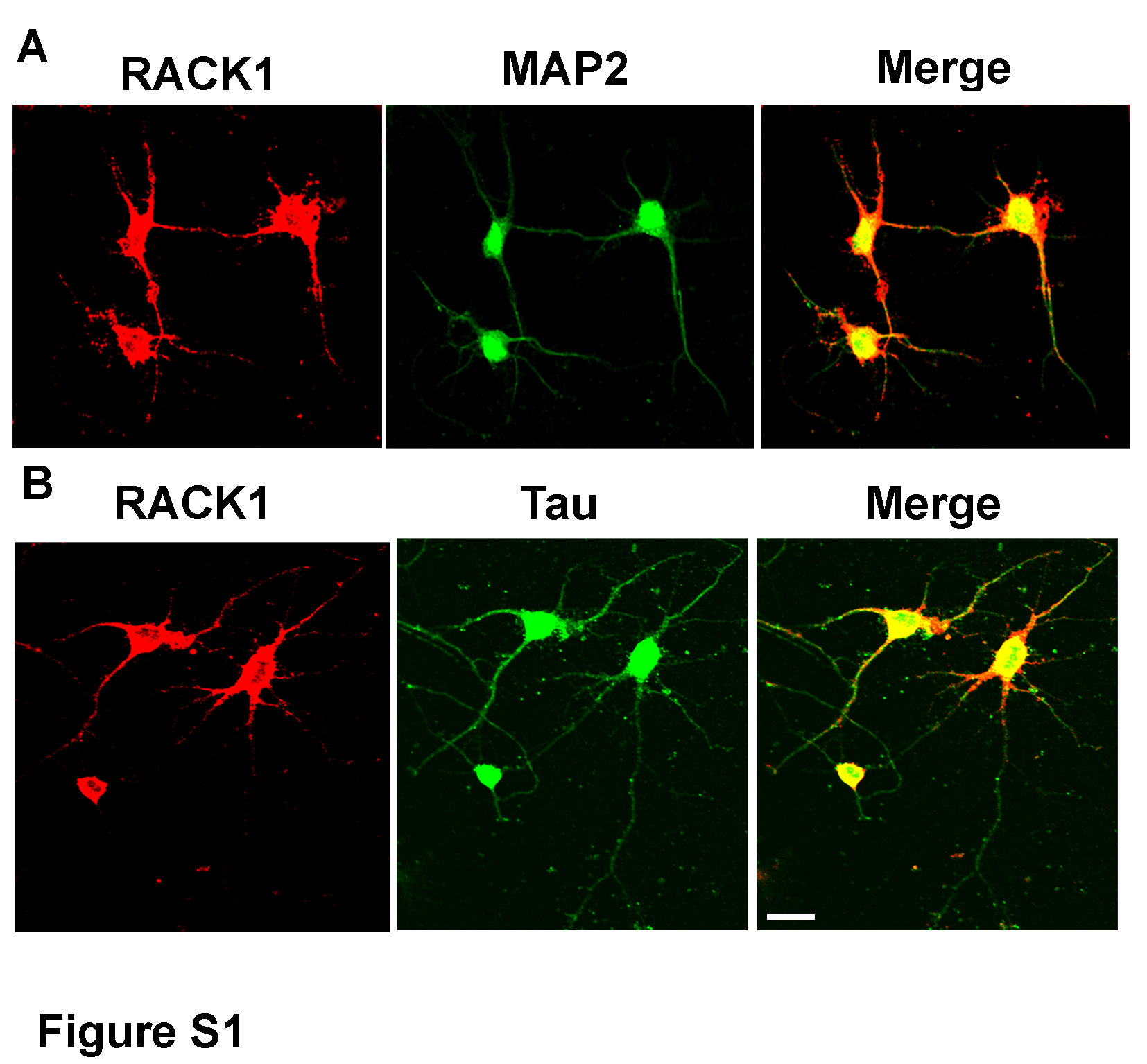

Supplement: Figure S1 — RACK1 along dendrites and axons of embryonic cortical cells. Co-immunostaining of MAP2 (A, green) or Tau (B, green) and RACK1 (red) indicated the localization of RACK1 in dendrites and axons. Scale bar 20 µm. (TIF) [file pone.0035034.s001.tif]

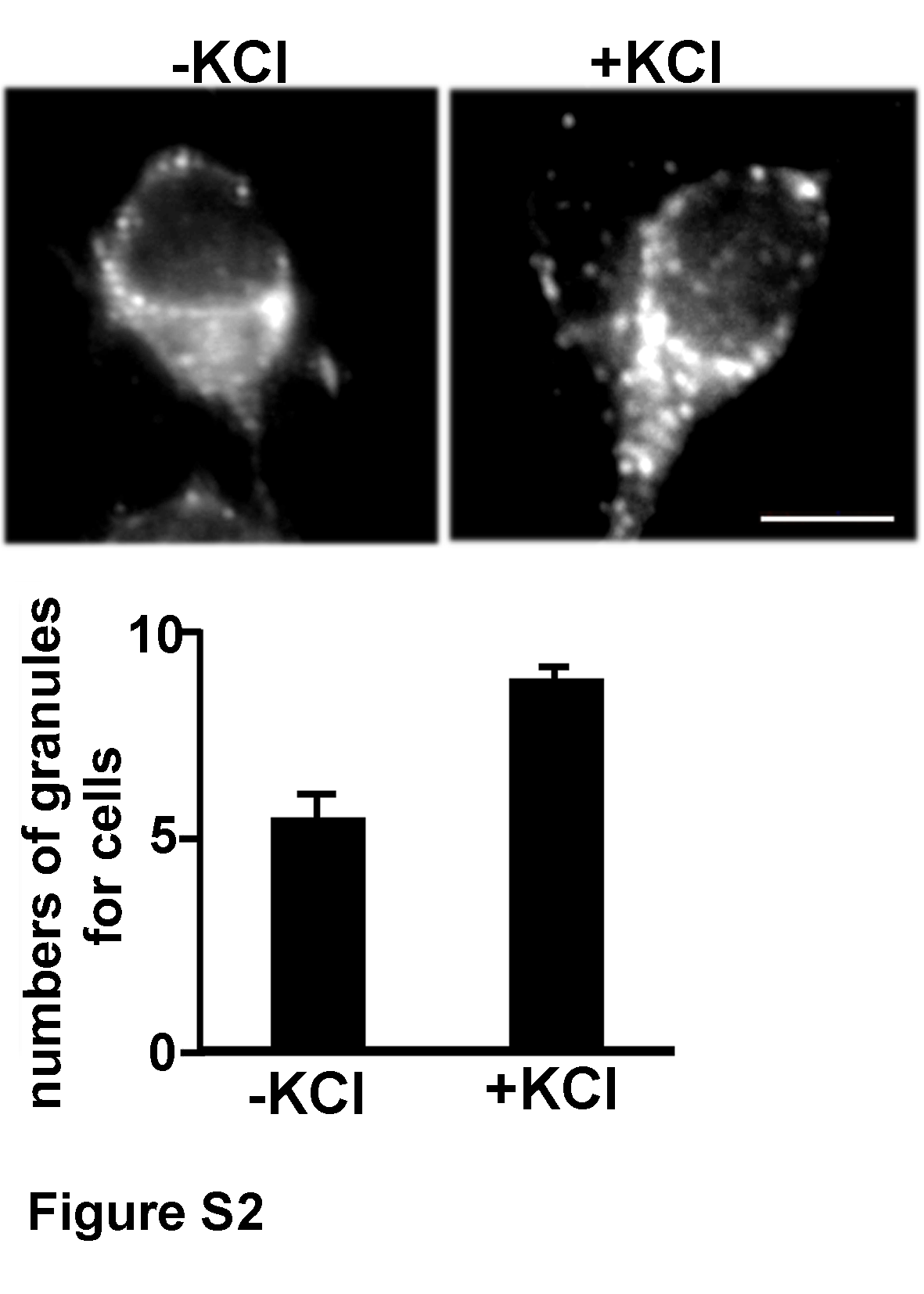

Supplement: Figure S2 — Neuronal activity reorganizes the distribution of RACK1. 50 mM KCl for 15 min increases the number of granules stained by RACK1. In the graphic are summarized the results observed in immunofluorescence in right Scale bar 20 µM. (TIF) [file pone.0035034.s002.tif]

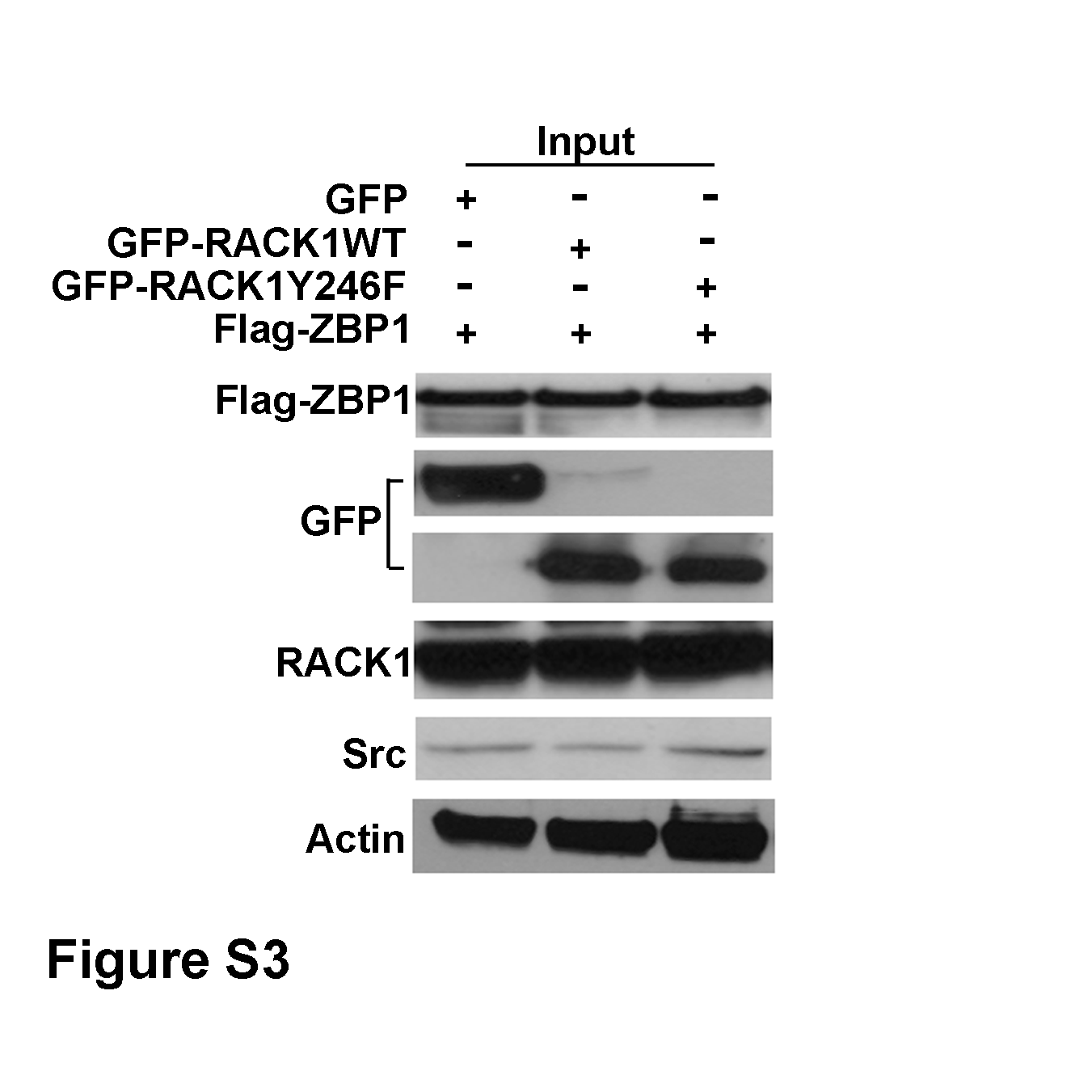

Supplement: Figure S3 — Transfection efficiency. Western blotting for GFP, GFP-RACK1, Flag-ZBP1, RACK1, Src and Actin on total lysate from SH-SY5Y cells co-expressing Flag-ZBP1 and GFP, GFP-RACK1wt or GFP-RACK1Y246F protein. (TIF) [file pone.0035034.s003.tif]

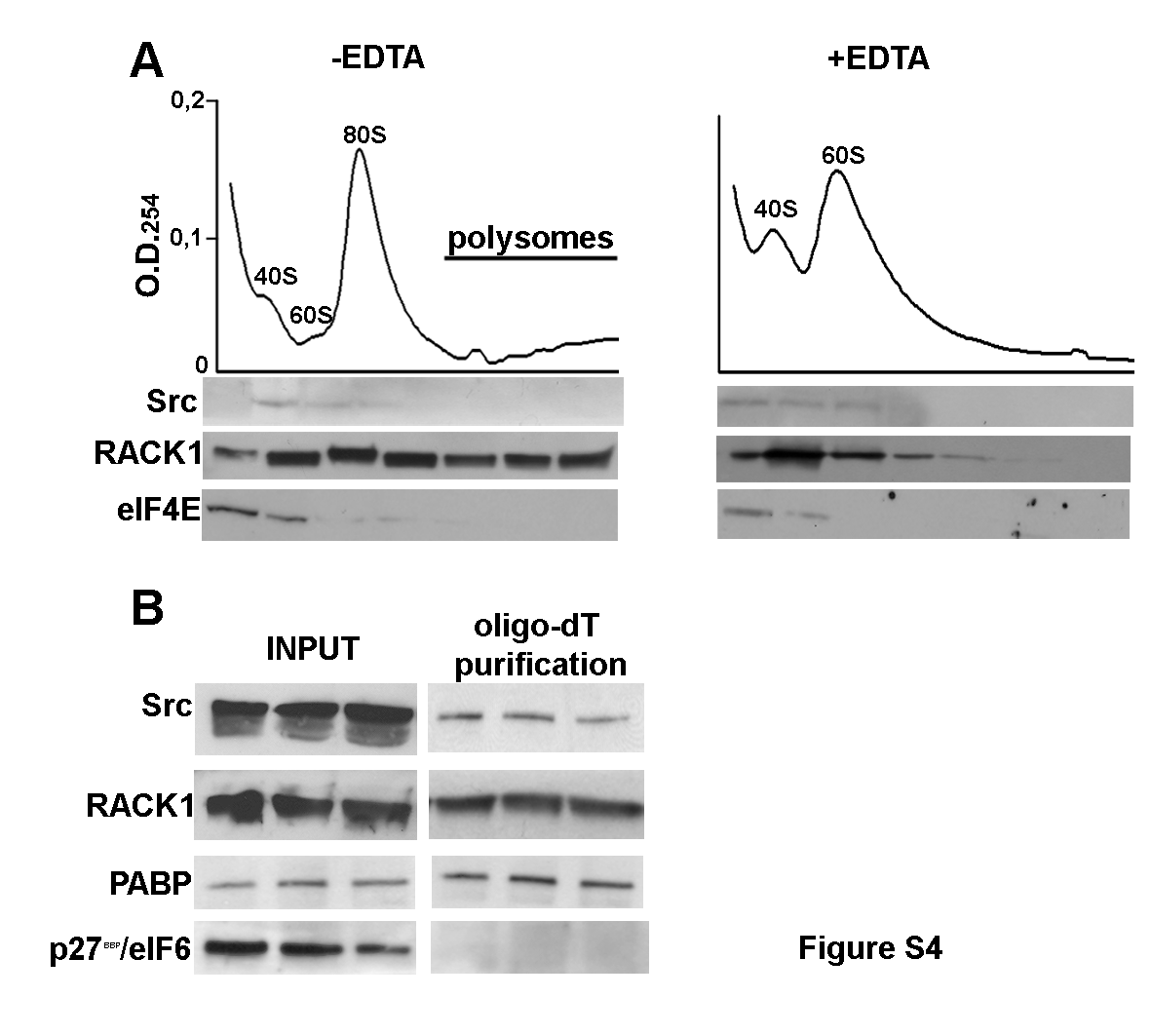

Supplement: Figure S4 — Src is part of the translational machinery. A, Src, as well as eIF4E and RACK1, localized in fractions at the top of the gradient, where 40S accumulated in the EDTA sucrose gradient from SH-SY5Y cells. B, Oligo-dT assay in cortical cells which specifically purifies proteins associated to mRNAs such as RNA binding proteins, 40S, 80S, polyribosomes and translational factors (ref 1 in Text S1). Src was specifically isolated as RACK1 and PABP proteins. p27BBP/eIF6, which binds only 60S ribosomal subunit and does not bind mRNAs, was not purified, indicating that the assay was specific for proteins associated to mRNAs. The figure shows the western blots on three independent experiments. (TIF) [file pone.0035034.s004.tif]

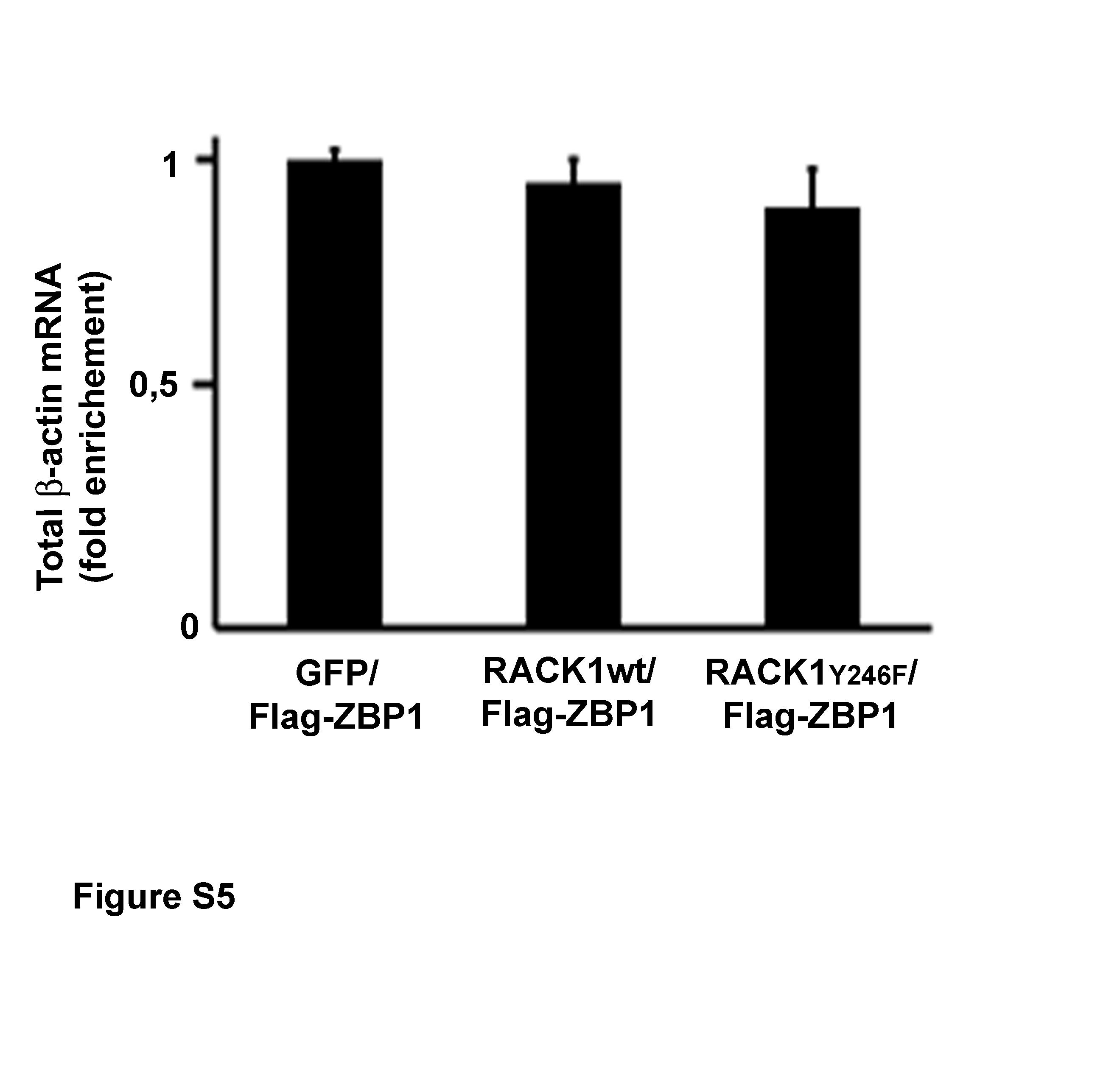

Supplement: Figure S5 — The β-actin mRNA expression in GFP-RACK1wt and GFP-RACK1Y246F overexpressing cells. Total β-actin mRNA, measured by qRT-PCR and normalized to 18S rRNA, is not affected by overexepression of GFP-RACK1wt and GFP-RACK1Y246F. (TIF) [file pone.0035034.s005.tif]
