# Supplementary material for: RACK1 Is a Ribosome Scaffold Protein for β-actin mRNA/ZBP1 Complex
Source: PLoS One. 2012 Apr 16;7(4):e35034. doi: 10.1371/journal.pone.0035034 (PMC3327689; doi:10.1371/journal.pone.0035034)
Supplement: Text S1 — (DOC) [file pone.0035034.s006.doc]

**Supporting information:**

**Oligo-dT purification**

Oligo-dT purification was performed in cortical neurons. The supernatant obtained as in ribosome profile was incubated with 50 μl oligo-dT cellulose (100 μg/ml, GE hEALTHCARE) for 2 hours at 4°C. The resins were extensively washed with 1 ml of polysomal buffer and eluted proteins were obtained adding 5X loading sample buffer and boiling [1].

**Production of GFP antibody**

EGFP cloned into the pQE plasmid (QIAgen) was used for recombinant GFP protein production from *E. coli*. The protein expression was carried out according to the manufacturer’s Instructions (*The QIAexpressionist 01/2000-Protocol 7* by QIAgen). The protein was purified through the His-tag by Affinity Chromatography, using the *Ni-NTA Agarose* (*QIAgen*).The purity of the protein was checked on SDS-PAGE and the protein was characterized via fluorescence spectroscopy. A polyclonal anti-GFP antibody was raised by rabbit immunization. For the immunization, BSA was used as a carrier for GFP. Cross-linking was achieved using glutaraldheyde, according to the protocol described in “Antibodies – A laboratory Manual” (1-2 peptide moles/50 aminoacids of carrier protein). The obtained GFP-BSA conjugated sample was dialyzed against PBS and used for a rabbit immunization (200 μg/immunizing injection).

The first bleeding was carried out after 31 days from the first antigenic injection. After 10 days, a second antigen injection was carried out. A total of four bloodlettings were done, at 10 days one from the other. The serum from the bloodlettings was tested using an ELISA assay, carried out in a direct format, on a plate coated with GFP. The serum was used as primary antibody.The purification of the anti-GFP antibody was achieved by affinity purification. The resin *CNBr-activated Sepharose 4B* (Pharmacia Biotech – GE) was conjugated with purified GFP according to manufacturer’s instructions. The conjugated resin was transferred into a separation column support and the purification was carried out using a peristaltic pump with a UV detector. The unconjugated groups on GFP were washed away with Tris-HCl 100 mM pH 8. Subsequently, the column was washed with 3 alternate pH cycles (acidic buffer: sodium acetate 100 mM pH 4, 500 M NaCl; basic pH buffer: 100 mM Tris-HCl pH 8, 500 mM NaCl).

The serum was purified on the column. The unbound sample was washed with 100 mM Tris-HCl pH 8 until a stable baseline. The antibodies were eluted by two subsequent elution step. The first one was an acidic elution, using 100 mM Glycine pH 2.5 (the collection tubes contained Tris-HCl pH 8.8 for pH neutralization). The column was then washed with 10 mM Tris-HCl pH 8.8. Finally, the basic elution was carried out using 100 mM triethylamine pH 11.5. The resin was finally regenerated with alternate pH cycles and saved in 0.05% NaN_3_/PBS at 4°C.

The eluted samples were tested in ELISA assay. The 96 wells ELISA plate (Falcon) was coated with increasing GFP concentration. As a negative control, BSA was also coated on the plate. The antibody was proven to have a good affinity for GFP

**Quantitive analysis of granules, FISH (Q-FISH), IF** **(QIF) and dendritic branching**

Q-FISH, Q-IF and immunofluorescence experiments were imaged on a Nikon Eclipse Ti microscope, in combination with a Nikon Intensilight excitation source and Photometrics Coolsnap HQ2 camera. Images were acquired with NIS-Elements software (Nikon) using a 60X objective (Nikon Plan Apo, N.A. 1.40) with the 1.5 multiplier built into the microscope (total magnification of 90X). In order to quantify fluorescent intensity, all experimental and image acquisition parameters were kept constant throughout each experiment.

In Q-FISH and Q-IF, we used a pseudocolor map to measure the fluorescent intensity. In false colour, black and blue indicated the lowest intensity pixels and increased through yellow to green, red, pink, and finally white (white indicates the highest intensity pixels). Using ImageJ, the fluorescent intensity was measured as the total fluorescent intensity in the growth cone and divided by the area to normalize for differences in growth cone size. This involved selecting a region of interest that encompassed the entire perimeter of the growth cone, including the central domain, lamellipodia and filopodia. In all experiments, the mean fluorescent intensity was background subtracted by measuring the mean fluorescent intensity in a region near, but not encompassing, the growth cone.

Two separate experiments were completed for each condition. In the first set, Flag-ZBP1 staining was performed to confirm that it was co-expressed with the GFP constructs. 20-30 cells per condition were examined (60-90 cells for the FISH experiments and 60-90 cells for the IF experiments), and GFP was co-expressed with Flag-ZBP1 in almost every cell. Only 1 cell did not show this co-expression, thus these two constructs are co-expressed more than 99% of the time. In the second set, staining was performed for β-actin (mRNA or protein), β-tubulin, and cells also expressed the GFP construct. Each experiment was repeated a minimum of three independent times. Appropriate statistical tests were performed using SPSS (IBM), depending on whether the data was parametric or non-parametric, and included one-way ANOVA, Kruskal-Wallis, Mann-Whitney, and Bonferroni tests. When significance was adjusted, it is referred to as alpha [2].

Dendritic branching out was measured by Sholl analysis. Briefly, we used the tracking function of NeruonJ plug-in of ImageJ software to draw a mask of all dendrites in GFP-fluorescence images. The mask was saved as separate image and used in automated Sholl plug-in of ImageJ software. Three separate experiments were completed and 30 cells were analyzed for each transfection type [3].

References

1. Napoli I, Mercaldo V, Boyl PP, Eleuteri B, Zalfa F, et al. (2008) The fragile X syndrome protein represses activity-dependent translation through CYFIP1, a new 4E-BP. Cell 134: 1042-1054.

2. Sasaki Y, Welshhans K, Wen Z, Yao J, Xu M, et al. Phosphorylation of zipcode binding protein 1 is required for brain-derived neurotrophic factor signaling of local beta-actin synthesis and growth cone turning. J Neurosci 30: 9349-9358.

3. Perycz M, Urbanska AS, Krawczyk PS, Parobczak K, Jaworski J Zipcode binding protein 1 regulates the development of dendritic arbors in hippocampal neurons. J Neurosci 31: 5271-5285.
